# Implementing healthy lifestyle promotion in primary care: a quasi-experimental cross-sectional study evaluating a team initiative

**DOI:** 10.1186/s12913-015-0688-4

**Published:** 2015-01-22

**Authors:** Kristin Thomas, Barbro Krevers, Preben Bendtsen

**Affiliations:** Department of Medical and Health Sciences, Linköping University, Linköping, Sweden; Department of Medical Specialist and Department of Medical and Health Sciences, Linköping University, Motala, Sweden

**Keywords:** Healthy lifestyle promotion, Primary care, Implementation fidelity, Coordination of care, RE-AIM framework

## Abstract

**Background:**

Non-communicable diseases are a leading cause of death and can largely be prevented by healthy lifestyles. Health care organizations are encouraged to integrate healthy lifestyle promotion in routine care. This study evaluates the impact of a team initiative on healthy lifestyle promotion in primary care.

**Methods:**

A quasi-experimental, cross-sectional design compared three intervention centres that had implemented lifestyle teams with three control centres that used a traditional model of care. Outcomes were defined using the RE-AIM framework: reach, the proportion of patients receiving lifestyle promotion; effectiveness, self-reported attitudes and competency among staff; adoption, proportion of staff reporting regular practice of lifestyle promotion; implementation, fidelity to the original lifestyle team protocol. Data collection methods included a patient questionnaire (n = 888), a staff questionnaire (n = 120) and structured interviews with all practice managers and, where applicable, team managers (n = 8). The chi square test and problem-driven content analysis was used to analyse the questionnaire and interview data, respectively.

**Results:**

Reach: patients at control centres (48%, n = 211) received lifestyle promotion significantly more often compared with patients at intervention centres (41%, n = 169). Effectiveness: intervention staff was significantly more positive towards the effectiveness of lifestyle promotion, shared competency and how lifestyle promotion was prioritized at their centre. Adoption: 47% of staff at intervention centres and 58% at control centres reported that they asked patients about their lifestyle on a daily basis. Implementation: all intervention centres had implemented multi-professional teams and team managers and held regular meetings but struggled to implement in-house referral structures for lifestyle promotion, which was used consistently among staff.

**Conclusions:**

Intervention centres did not show higher rates than control centres on reach of patients or adoption among staff at this stage. All intervention centres struggled to implement working referral structures for lifestyle promotion. Intervention centres were more positive on effectiveness outcomes, attitudes and competency among staff, however. Thus, lifestyle teams may facilitate lifestyle promotion practice in terms of increased responsiveness among staff, illustrated by positive attitudes and perceptions of shared competency. More research is needed on lifestyle promotion referral structures in primary care regarding their configuration and implementation.

## Background

Many non-communicable diseases (NCDs), for example, cardiovascular diseases, cancers and diabetes, account for about two-thirds of all deaths worldwide [1,2]. Health-enhancing behaviours such as tobacco cessation, physical activity, a balanced diet and moderate alcohol consumption could prevent 80% of coronary heart disease, 90% of type 2 diabetes, and 30% of all forms of cancer [3]. Health care organizations are therefore encouraged to integrate the promotion of healthy lifestyles in routine practice in order to reduce the burden of NCDs [4]. The primary care sector is suggested as a suitable setting for lifestyle promotion because of its capacity to reach a large proportion of the population, its credibility, continuity of care and it is the first point of contact for many patients [5,6]. In Sweden, a health-promoting health care has been further supported by national public health policies and the release of national guidelines for lifestyle promotion in health care [7,8]. Similar developments are taking place internationally [9-12].

However, re-orienting primary care to include routine healthy lifestyle promotion is challenging [13]. The proportion of patients receiving lifestyle advice varies between a few percent to about 30% [14-16]. A study investigating video recordings of consultations in Dutch general practices between 1975 and 2008 showed that only 6–13% of consultations included lifestyle advice [15]. Furthermore, barriers for integrating lifestyle promotion in primary care have been shown to be intrapersonal (perceived effectiveness of interventions, beliefs, attitudes, motivations and confidence), interpersonal (patient characteristics or lack of cooperation with other disciplines) and institutional (time and referral resources) [5,17-21]. Strategies to overcome barriers such as audits and feedback, education or reminders have had minor effects [22,23]. Taxonomies have been developed to evaluate the impact of strategies on practice outcomes [23,24]. For example, it has been proposed that coordination of health services and increased collaboration between professionals within primary care could help to overcome implementation barriers [5,24]. However, research has shown that collaboration in primary care practices can be challenging due to conflicts between profession groups, slow-moving decision making and lack of understanding of each other’s roles, knowledge and responsibilities [25-28]. Furthermore, implementing effective strategies in routine practice under real-world conditions has been found to be challenging and require more research [29,30].

Thus, more knowledge is needed regarding how coordination of care is implemented in routine primary care and subsequently how lifestyle promotion may be facilitated through these initiatives [24,31]. This article reports on an evaluation of a real-world lifestyle team initiative implemented in primary care in southeast Sweden. These teams aimed to facilitate healthy lifestyle promotion practices through increased collaboration and coordination of care. Healthy lifestyle promotion entailed lifestyle counselling (screening or giving advice) and referral of patients to staff specialized in lifestyle promotion at the primary care centre. Primary care centres in Sweden consist of physicians, dieticians, behavioural therapists and specialist nurses. The lifestyle team initiative aimed to use the existing multi-professional structure of Swedish primary care. Similar models have been used successfully for diabetes care. For example, patients with increased glucose levels are referred to specialist diabetic nurses [32]. This study aims to evaluate the impact on healthy lifestyle promotion of a lifestyle team initiative. The RE-AIM framework was used to identify outcome variables [33]. Four RE-AIM components were investigated: reach (proportion of patients receiving lifestyle promotion); effectiveness (attitudes and competency among staff); adoption (lifestyle promotion practice among staff); implementation (fidelity to the lifestyle team protocol). RE-AIM was chosen as it allowed for evaluating impact and implementation simultaneously (Table 1 show original RE-AIM definitions and the definitions used in this study).

**Table 1:** **Original and current study definitions of RE-AIM dimensions**

**Dimension**	**Original definitions**	**Current study**		
		**Definition**	**Variable**	**Measurement**
Reach	The absolute number, proportion and representativeness of individuals who are willing to participate in a given initiative	The proportion of patients who received healthy lifestyle promotion in the last 6 months	Proportion of patients	National patient survey
Effectiveness	The impact of an intervention on important outcomes, including potential negative effects, quality of life, and economic outcomes	Attitudes and competency among staff regarding healthy lifestyle promotion and the lifestyle teams	Proportion of staff agreeing with attitude and competency statements	Staff questionnaire
Adoption	The absolute number, proportion, and representativeness of settings and intervention agents who are willing to initiate a program	Proportion of staff who daily engage in healthy lifestyle promotion practice	Proportion of staff reporting daily healthy lifestyle promotion practice	Staff questionnaire
Implementation	At the setting level, implementation refers to the intervention agents’ fidelity to the various elements of an intervention’s protocol	Fidelity to the lifestyle team protocol: Team structure, coordinator, referral structure, regular meetings	Implementation fidelity	Interviews with practice managers and team managers
Maintenance	The extent to which a program becomes part of the routine organizational practices	Not included in the study		

## Methods

### Study design

A quasi-experimental, cross-sectional design compared two groups of primary care centres: three intervention primary care centres that had been commissioned to implement lifestyle teams and three control centres that used a traditional model of care. Outcome variables were defined using the RE-AIM framework (Table 1). Data collection methods included a patient questionnaire, a staff questionnaire and structured interviews with managers.

### The lifestyle team initiative

The lifestyle team initiative was instigated by a regional primary care management group in Östergötland County Council and developed in dialogue with primary care staff in the area. The lifestyle team concept grew from an analysis of current practice, barriers and resources for integrating lifestyle promotion at primary care centres. The following barriers were identified: scarce time and resources, negative attitudes among staff towards lifestyle promotion and lack of a clear lifestyle promotion patient pathway at the centres. The aim was to overcome these barriers through improved collaboration and coordination regarding lifestyle promotion. The lifestyle team protocol stated that centres should have (1) a multi-professional structure, (2) a team coordinator, (3) team meetings at least every 6 weeks and (4) an explicit in-house referral structure for patients with health risk behaviours (e.g. sedentary lifestyle, risky alcohol consumption, poor nutrition or tobacco consumption). How the work was organized beyond the four components and any potential implementation strategies were left to each centre to define e.g., how to refer a patient or meeting agendas. No financial support or materials was given aimed to facilitate implementation of the teams. Both intervention and control centres have been given training in methods for lifestyle promotion, e.g., motivational interviewing.

### Setting

Östergötland County Council is divided into four primary care subregions each with their own primary care management group. The intervention and control centres operated in two of these subregions (western and central) but were bound by similar financial and budgetary constraints; they were comparable regarding size, setting and socioeconomic factors. About 26 700 and 26 000 patients were listed at the intervention and control centres, respectively (according to County Council database, 2011). Randomization was not feasible as the lifestyle teams were already in place when data collection commenced. The county council has a history of working towards a health-promoting health service. All the centres (intervention and control) had similar availability of health care professions. All centres had in-house access to dieticians, physiotherapists, occupational therapists, behavioural therapists and specialized nurses. The intervention centres aimed to coordinate these professions by implementing the lifestyle teams (e.g., through meetings and coordination).

#### Intervention centres

In December 2008, all ten primary care centres in the western sub-region of Östergötland County Council were commissioned to implement lifestyle teams. Three of the ten centres were invited to take part in this project. A best-practice inclusion criterion was applied based on a status update report carried out by the regional primary care management group. The report showed that the three centres had started implementing lifestyle teams at the time of recruitment, which made them suitable for inclusion in the study. The centres were situated in one urban setting.

#### Control centres

The three control centres were located in the central subregion. None of the control centres had been commissioned to implement lifestyle teams. Control centres comparable with the intervention centres in terms of size (listed patients) and setting and within the same county council were selected. Control centres were also situated in one urban setting. Control centres used regular care of lifestyle promotion including following national and local guidelines, e.g., giving brief advice in general practice when deemed necessary.

### Participants and data collection

#### Reach: patients

Extracted data from a national patient survey was used to measure reach at the six participating centres. Computerized random sampling from a patient register was used to identify potential participants. Patients who had visited the primary care centres during September 2011 were included in the register. For each centre, 300 invitations were sent by post; 200 of patients had visited a physician and 100 a nurse. Patients could choose to complete the survey on paper or online. Inclusion criterion for the current study was age 16 years or older.

#### Effectiveness and adoption: health care staff

The staff questionnaire used to measure effectiveness and adoption was distributed via e-mail in September 2011. An e-mail including information about the study and a link to the questionnaire was sent to all staff members who had individual patient contact at the six centres (n = 165). Two reminders were sent via e-mail 2 and 3 weeks after the initial e-mail. Physicians, nursing professions, dieticians and behavioural therapists were included.

#### Implementation: practice managers and team managers

Implementation fidelity was investigated using telephone interviews with managers during October 2011. All practice managers and all lifestyle team managers at the intervention and control centres were invited to take part in the interviews (n = 8). Interviews were carried out with all centres to verify that the lifestyle teams were or were not implemented. The interviews lasted for about 30 minutes and participants could select a suitable time for the interview. Notes were taken during the interviews using the interview guide as a response sheet to aid accuracy. All interviews were carried out by K.T.

### Outcome variables and application of the RE-AIM framework

Four RE-AIM components were investigated: reach (proportion of patients receiving lifestyle promotion); effectiveness (attitudes and competency among staff); adoption (lifestyle promotion practice among staff); implementation (fidelity to the lifestyle team protocol) [33]. Healthy lifestyle promotion was measured through the reach of patients and adoption among staff to capture both perspectives. Implementation refers to fidelity to the team protocol rather than treatment fidelity of clinical practice [34]. Definitions of the RE-AIM elements are presented in Table 1.

### Measures

#### Reach: patient questionnaire

Data from a Swedish national patient survey [35] were used to assess the proportion of patients who received lifestyle promotion. The patient survey is administered by Swedish association of Local Authorities and Regions and distributed bi-annually to primary care centres in Sweden. The overall aim of the survey was to investigate quality of care, patient participation and care accessibility in primary care. The patient data used in this study was extracted from the national data set. One item was used in the current study: “Did the physician or other staff discuss [lifestyle behaviour] with you?” The item was repeated for eating habits, physical activity, tobacco and alcohol consumption. For each lifestyle behaviour there were three response options: (1) Yes, at the current visit, (2) Yes, at a visit during the last 6 months, and (3) No. Dichotomized response options were used as the primary outcome for reach; responses (1) and (2) were analysed as patients having received lifestyle promotion. Two items investigating age and gender were used to investigate responder characteristics.

#### Effectiveness and adoption: staff questionnaire

The questionnaire was generated by the research team, based on a thorough review of the research literature, reviewed by an expert panel and pilot tested among target groups. Items were subsequently modified within the research group to capture the aim of the study and to achieve face and content validity.

Effectiveness was assessed using self-reported attitudes and competency regarding lifestyle promotion practice and the lifestyle teams. Eight items were used with a four-point response scale (from 1 = strongly agree to 4 = strongly disagree) and the alternative “do not know” (see Table 2 for details of the items).

**Table 2:** **Effectiveness comparison: number and percentage of staff agreeing with attitude and competency statements**

	**Intervention centres (** ***n*** **= 77)**		**Control centres (** ***n*** **= 43)**		***P-value*** ^**1**^	***P-value*** ^**2**^ ***adj. by center***
	**n/N**	**(%)**	**n/N**	**(%)**		
**Self-reported attitude**						
There is a need for a lifestyle team or similar initiative at my centre	67/73	(92)	30/39	(77)	0.028^a^	0.026
It is important that primary care centres offer support regarding healthy living	69/72	(96)	38/39	(97)	1.000^b^	0.699
Lifestyle counselling is an efficient method to support patients in behaviour change	70/70	(100)	33/37	(89)	0.013^b,3^	−^†^
Issues regarding healthy lifestyle promotion are prioritized at my centre	36/69	(52)	7/35	(20)	0.002^a,3^	<0.001^3^
**Self-reported competency**						
I have sufficient competency to give patients lifestyle advice	65/73	(89)	38/41	(93)	0.744^b^	<0.001^3^
During a typical consultation I have sufficient time to discuss healthy living with patients	38/73	(52)	15/40	(38)	0.138^a^	0.085
There is sufficient competency (knowledge, skills) at my centre to manage issues regarding healthy lifestyle promotion	69/70	(99)	31/38	(82)	0.003^b,3^	0.002^3^
Sometimes it is uncomfortable to bring up healthy living with patients	22/73	(30)	13/40	(33)	0.795^a^	0.760

^1^Univariate comparisons by ^a^chi-squared test or ^b^Fisher’s exact test.

^2^Model I: adjusted for the effects of cluster allocation.

^†^P-value can not be estimated (due to lack of variation in the intervention group).

^3^ *P* < 0.05/4 = 0.013 (with Bonferroni adjustment).

Adoption, that is, self-reported lifestyle promotion practice, was investigated using two items: (1) “How often do you ask patients about their lifestyle behaviours (physical activity, eating habits, and tobacco or alcohol consumption?” and (2) “How often do you refer patients to staff specialized in lifestyle promotion”. Response options for both items were (1) daily, (2) once/several times a week, (3) once/several times a month, (4) less often, and (5) never. Adoption of lifestyle promotion was defined as daily practice, however weekly practice is also reported. Three items measured responder characteristics: age, gender and profession.

#### Implementation fidelity: individual interviews

A structured interview guide was used based on the lifestyle team protocol. It included four close-ended questions representing the four components of the protocol: is there a multi-professional team, a team coordinator, an explicit in-house referral structure and are regular team meetings being held? A further eight open-ended questions aimed to explore the degree of fidelity regarding the teams (size, professions included and what was discussed at meetings), team development (the definition, review and dissemination of team goals) and the referral structure (dissemination and use among staff).

### Data collection procedure

#### Patients

The national patient survey is distributed bi-annually to a random sample of patients. For the 2011 survey, patients who had visited their primary care centre (their physician or nurse) during September were invited to complete the survey. For each centre, 300 invitations were sent by post. Patients could choose to complete the survey on paper or online. For the current study, data for the six participating centres were extracted from the national dataset.

#### Health care staff

The staff questionnaire used to measure effectiveness and adoption was distributed via e-mail in September 2011. An e-mail including information about the study and a link to the questionnaire was sent to all staff members who had individual patient contact at the six centres. Two reminders were sent via e-mail 2 and 3 weeks after the initial e-mail.

#### Practice managers and team managers

Implementation fidelity was investigated using telephone interviews with practice managers and team managers during October 2011. Interviews were carried out with all centres to verify that the lifestyle teams were or were not implemented. The interviews lasted for about 30 minutes and participants could select a suitable time for the interview. Notes were taken during the interviews using the interview guide as a response sheet to aid accuracy. All interviews were carried out by K.T.

### Data analysis

Differences between proportions were tested by chi-squared test or Fisher’s exact test in case of small sample sizes.

The binary outcomes of reach in Table 3 were compared between groups with logistic regression adjusted for age, sex, and type of visit (physician or nursing profession) using robust standard errors to take account of clustering effects within each primary health care centre (model II). Bonferroni adjustment for multiple end points was applied in the analyses of differences in effectiveness items; attitude and competency groups of items.

**Table 3:** **Reach comparison: number and percentage of patients who received healthy lifestyle promotion**

	**Intervention (** ***n*** **= 433)**		**Control (** ***n*** **= 455)**		**P-value**
	***n/N***	**(%)**	***n/N***	**(%)**	
Eating habits					
Current visit	54/411	(13)	63/439	(14)	0.795
Last 6 months	41/411	(10)	53/439	(12)	0.556
Total^2^	95/411	(23)	116/439	(26)	0.522
Physical activity					
Current visit	71/403	(18)	79/433	(18)	0.835
Last 6 months	46/403	(11)	76/433	(18)	<0.001
Total^2^	117/403	(29)	155/433	(36)	0.035
Tobacco consumption					
Current visit	70/402	(17)	82/428	(19)	0.470
Last 6 months	39/402	(10)	39/428	(9)	0.682
Total^2^	109/402	(27)	121/428	(28)	0.650
Alcohol consumption					
Current visit	49/406	(12)	48/432	(11)	0.224
Last 6 months	30/406	(7)	36/432	(8)	0.808
Total^2^	79/406	(19)	84/432	(19)	0.648
Lifestyles combined					
Current visit	110/416	(26)	140/441	(32)	0.187
Last 6 months	74/416	(18)	101/441	(23)	0.096
Total^2^	169/416	(41)	211/441	(48)	0.024

^2^Current visit and visit in last 6 months combined.

For implementation fidelity, interview data were analysed using deductive problem-driven content analysis [36]. The analysis was based on the lifestyle team protocol whereby data describing the implementation of the four components were identified and synthesized. Both the implementation (close-ended questions) and degree of fidelity (open-ended questions) of the components were investigated.

### Ethical approval

The study was conducted with the approval of the Regional Ethical Review Board in Linköping (DNR: IMH-2009-00335).

## Results

### Response rates

#### Patients

A total of 888 eligible responders were included in the current study. Details on gender, age and visit (physician or nurse) for the sample included in the analysis are shown in Table 4.

**Table 4:** **Patient sample data: age, gender and visit characteristics** ^**1**^

	**Response rate,** ***n/N*** **(%)**			**P value** ^**a**^
	**Intervention**	**Control**	**Total**	
Gender				
Women	251/424 (59)	282/449 (63)	533/873 (61)	0.275
Men	173/424 (41)	167/449 (37)	340/873 (39)	
Age				
16–44 years	70/416 (17)	122/447 (27)	192/863 (22)	0.003
45–65 years	136/416 (33)	136/447 (30)	272/863 (32)	
65–74 years	97/416 (23)	87/447 (20)	184/863 (21)	
75+ years	113/416 (27)	102/447 (23)	215/863 (25)	
Type of visit				
Physician	276/433 (64)	307/455 (67)	583/888 (66)	0.242
Nursing profession	157/433 (36)	148/455 (33)	305/888 (34)	

^1^Randomized sample of patients who visited their primary care centre during September 2011.

^a^Chi-squared test.

#### Health care staff

In total, 120 (78%) responded to the staff questionnaire: 77 (78%) at intervention centres (83% females) and 43 (65%) at control centres (85% females). The mean age was 48 years (SD 11 years) and 47 years (SD 11 years) for the intervention and control centres, respectively. Nursing professions (63%) represented the largest group of responders for both groups followed by physicians (28%) and allied health care professions (9%).

#### Practice managers and team managers

All six practice managers and two team managers took part in telephone interviews to investigate implementation. At one of the intervention centres, the practice manager and the team coordinator was the same person. All were women with a mean age of 57 years (SD 2 years).

### Main results

#### Reach

For lifestyle behaviours combined, significantly more patients at control centres (48%) received lifestyle promotion compare with intervention centres (41%) (see Table 3 for the results for combined and separate lifestyle behaviours). This difference remained for physicians but not nurses when visits were assessed separately: 51% at control centres compared with 42% at intervention centres received lifestyle promotion when visiting their physician. These figures were 38% at intervention centres and 42% at control centres for nurse visits. Analyses for each lifestyle behaviour showed that intervention and control centres differed significantly regarding promotion of physical activity: 36% of patients at control centres compared with 29% at intervention centres. These differences applied to both nurse and physician visits. In general, promotion of moderate alcohol consumption occurred less often than promotion of the other healthy lifestyles.

#### Effectiveness

Intervention and control centres differed significantly on three of the eight items on self-reported attitude and competency concerning lifestyle promotion practice. Intervention staff was significantly more likely to agree that lifestyle promotion was an efficient method, their centre had sufficient competency and that lifestyle promotion was prioritized at their centre (see Table 2 for results for each item).

#### Adoption

No differences on adoption of healthy lifestyle promotion among staff was found; 47% (*n* = 34) of staff at intervention centres and 58% (*n* = 24) at control centres reported that they asked patients about their lifestyles on a daily basis. Eighty-eight percent of physicians (intervention, 81%; control, 94%) and 34% of nursing and allied health care (intervention, 35%; control, 29%) reported asking patients daily. Most staff (intervention, 89%; control, 88%) reported that they gave lifestyle promotion at least once a week.

Both intervention and control staff referred patients to other professions at the health care centre who specialized in lifestyle promotion; 27% (*n* = 20) at intervention centres and 31% (*n* = 13) at control centres did this on a weekly basis. Fifty-eight percent of physicians (intervention, 50%; control, 64%) and 20% of nursing and allied health care (intervention, 25%; control, 10%) referred patients on a weekly basis. Only one staff member at an intervention centre and two at control centres reported daily referral practice.

#### Implementation fidelity

All centres had implemented teams with a multi-professional structure, that is, practice managers, dieticians, physicians, behavioural therapists, medical secretaries and varied nursing professions (nurses specializing in diabetes, asthma and lifestyle). All teams had managers and held team meetings at least every 6 weeks. Team meetings were used to evaluate (e.g. referral rates), plan future projects (e.g. open house days) and manage ongoing activities (e.g. updating patient information in the waiting area). One centre had developed an explicit in-house referral structure for patients with health risk behaviours. The other two centres expressed that there was an understanding among staff of how, and to whom, to refer patients. All intervention centres expressed that the referral structure was used inconsistently for patients with risk behaviours only, that is, for a primary prevention purpose. No specific goals had been defined for the teams. Two centres referred to health promotion goals set by the County Council and stated that the lifestyle team was a vehicle to reach these goals. Control centres had not implemented any lifestyle teams.

## Discussion

We set out to evaluate the impact of a lifestyle team initiative on healthy lifestyle promotion practice in primary care. Differences on both effectiveness (i.e. attitudes and self-reported competency) and reach (i.e. practice behaviour) were found between the groups. All intervention centres had implemented multi-professional teams, appointed team managers and held regular meetings but struggled to implement a referral structure that was used consistently among staff.

### Reach

The intervention and control centres did not differ on the frequency of promotion of any lifestyle behaviour except for physical activity, where control centres had higher rates. The findings may be explained by individual practitioner factors (e.g., attitudes) as well as contextual factors (e.g., social support or norms) [37]. Attitudes among staff regarding a specific practice have been shown to facilitate its adoption [38,39]. Most of the staff at both intervention and control centres had positive attitudes and self-reported competency, indicating good conditions for implementing lifestyle promotion at these centres. Contextual factors such as exercise referral schemes and associated financial incentive programmes can facilitate the promotion of physical activity [40]. However, both intervention and control centres were exposed to similar strategies at the county council level. Furthermore, the long-term impact of these types of strategies is unclear [41,42].

### Effectiveness

As previously stated, the findings indicated good pre-conditions for healthy lifestyle promotion at both intervention and control centres in terms of positive attitudes and self-reported competency among staff [39]. The intervention centres showed additional facilitating factors; staff was more positive towards the effectiveness of lifestyle counselling, shared competency and how lifestyle promotion was prioritized at their centre. Thus, barriers for lifestyle promotion may have been somewhat reduced. The lifestyle teams may have promoted a sense of shared competency among staff through the coordination of competencies and prioritization of lifestyle promotion at the centres. However, the lifestyle teams, in terms of being a referral resource, did not succeed in removing lack of time as a barrier for practice change. Even though staff at intervention centres more often agreed that they had sufficient time to discuss lifestyle with patients, the groups did not differ significantly. It is unclear how much of a barrier time is for lifestyle promotion. A randomized control trial showed that lifestyle counselling caused little or no increase in the duration of a routine visit in primary care [43]. Thus, the conceptual differences between the study groups may indicate initial, albeit important, conceptual changes, which require longer follow-up to determine their impact on long-term practice change [44]. Most staff agreed that there was a need for a lifestyle team, or similar initiative, at their centre, which indicates a need for practice models in primary care to facilitate lifestyle promotion.

### Adoption

There were no differences between the groups on adoption. A potential ceiling effect may explain the lack of difference as about 90% of physicians claimed that they asked patients about their lifestyles on a daily basis. It is unclear what constitutes the optimal rate of lifestyle promotion in primary care. Studies show that both patients and practitioners think lifestyle promotion is important in primary care [45]. Furthermore, the target group for lifestyle promotion could be substantial in Sweden as almost every second Swede engages in at least one health-compromising behaviour [46]. However, a 100% lifestyle promotion rate would not be reasonable as lifestyle promotion is only one of many practices that need to be prioritized in a routine visit [47]. It is important to establish expectations and practice goals that are both reasonable to implement in routine primary care and still meet public health needs. One way to make healthy lifestyle promotion practice more efficient could be the use of in-house referral resources.

Intervention and control staff showed comparable internal referral behaviour rates; about a third of staff claimed to do this on a weekly basis. Limited referral behaviour is consistent with most staff reporting feeling competent in lifestyle promotion, which would make referral to a colleague redundant. However, coordinated care models (e.g. diabetes care) have been shown to be a beneficial resource acknowledging the competency of multiple professions and that the collaboration between them is important for patient outcomes. Referral to in-house resources, compared with external resources, has been found to facilitate referral behaviour. Findings have been explained by familiarity with the resource and close collaboration opportunities [17,24]. All intervention centres struggled to implement working referral structures for lifestyle promotion, a resource that could have facilitated referral practice further. More research is needed on lifestyle promotion referral structures or patient pathways in primary care regarding their configuration and implementation.

### Implementation

The aim of the lifestyle teams was to improve the collaboration and coordination of lifestyle promotion practices. It is uncertain to what extent this was achieved at the intervention centres. The original protocol included predominantly structural components, that is, appointment of team managers rather than process components on how to collaborate. These structural changes were perhaps not sufficient to facilitate coordination and subsequently lifestyle promotion practice. In addition, intervention centres struggled to implement a referral structure that was used consistently among staff, which could have reduced opportunities for coordinated care. Lack of team goals might indicate that the teams were at an early phase of their group development. Factors such as shared goals and shared team identity have been found to be important for team work in primary care [28].

Our study shows that there may be implementation challenges specific to lifestyle team initiatives. Implementing the teams involves change across several systems at structural, behavioural and interpersonal levels, which has been found to be challenging [48]. Furthermore, multi-professional compared with profession-specific change has been found to be more difficult to implement [44]. Profession-specific understanding of teamwork was identified in a qualitative study where a directive philosophy (mostly held by medical professions) was characterized by hierarchy, status and elected team leaders [49]. Thus, these aspects need to be considered when implementing coordinated care models in the future.

### Limitations

Cross-sectional studies are always restricted in causal inference. Due to logistical constraints relating to the timing of the evaluation, it was not possible to include pre-test data of variables. However, the intervention and control centres were suitable for comparison as they were homogeneous regarding: lifestyle promotion guidelines, financial and budgetary constraints, size and setting. Also, even though the teams were described as important vehicles for continuous improvement, impact on reach, effectiveness and adoption may require longer follow-up including the implementation of working referral structures as discussed earlier.

## Conclusions

Intervention centres did not show higher rates than control centres on reach of patients or adoption among staff at this stage. All intervention centres struggled to implement working referral structures for lifestyle promotion. The protocol was predominantly structural and may not have been sufficient to facilitate implementation of the teams and subsequent change in practice behaviour. Intervention centres were more positive on effectiveness outcomes, attitudes and competency among staff, however and the teams were perceived to be an important component at the centres. Thus, in the long term, the lifestyle teams may facilitate healthy lifestyle promotion practice in terms of increased responsiveness among staff illustrated by positive attitudes and perceptions of shared competency. More research is needed on lifestyle promotion referral structures in primary care regarding their configuration and implementation.
